# Molecular Evolution of a Peptide GPCR Ligand Driven by Artificial Neural Networks

**DOI:** 10.1371/journal.pone.0036948

**Published:** 2012-05-14

**Authors:** Sebastian Bandholtz, Jörg Wichard, Ronald Kühne, Carsten Grötzinger

**Affiliations:** 1 Charité – Universitätsmedizin Berlin, Campus Virchow-Klinikum, Department of Hepatology and Gastroenterology and Molecular Cancer Research Center (MKFZ), Tumor Targeting Lab, Berlin, Germany; 2 Leibnitz-Institut für Molekulare Pharmakologie (fmp), Berlin, Germany; Charité-Universitätsmedizin Berlin, Germany

## Abstract

Peptide ligands of G protein-coupled receptors constitute valuable natural lead structures for the development of highly selective drugs and high-affinity tools to probe ligand-receptor interaction. Currently, pharmacological and metabolic modification of natural peptides involves either an iterative trial-and-error process based on structure-activity relationships or screening of peptide libraries that contain many structural variants of the native molecule. Here, we present a novel neural network architecture for the improvement of metabolic stability without loss of bioactivity. In this approach the peptide sequence determines the topology of the neural network and each cell corresponds one-to-one to a single amino acid of the peptide chain. Using a training set, the learning algorithm calculated weights for each cell. The resulting network calculated the fitness function in a genetic algorithm to explore the virtual space of all possible peptides. The network training was based on gradient descent techniques which rely on the efficient calculation of the gradient by back-propagation. After three consecutive cycles of sequence design by the neural network, peptide synthesis and bioassay this new approach yielded a ligand with 70fold higher metabolic stability compared to the wild type peptide without loss of the subnanomolar activity in the biological assay. Combining specialized neural networks with an exploration of the combinatorial amino acid sequence space by genetic algorithms represents a novel rational strategy for peptide design and optimization.

## Introduction

G protein-coupled receptors (GPCRs) regulate vital cellular functions such as energy and ion homeostasis, cellular adhesion, motility and also proliferation [1], [2]. For their involvement in many physiological processes relevant in diseases ranging from diabetes to cancer, GPCRs are considered one of the most valuable classes of protein targets on the cell membrane [2], [3]. At least one third of all currently marketed drugs are directed against GPCRs, while due to the lack of highly potent and stable ligands many other receptors of this protein superfamily still await their pharmaceutical use [4]. In this target class, structure-based drug discovery using rational design is still hampered by the small number of available 3D data for GPCRs. When this study was initiated only five x-ray structures of GPCRS were known: those of of two rhodopsins (PDB 1F88, 2Z73) [5], [6], of the β2- and β1-adrenergic receptors (PDB 2RH1, 2VT4) [7], [8] and the structure of the A2A adenosine receptor (PDB 2RH1) [9]. Within the last two years the structures of the CXC chemokine receptor type 4 (PDB 3OE0, 3ODU) [10], dopamine D3 receptor (PBD 3PBL) [11] and the histamine H1 receptor (PDB 3RZE) [12] were determined. Thus, CXCR4 is the only peptide/protein ligand GPCR with a known three-dimensional structure so far. Consequently, alternative approaches for molecular design of potential drugs are being explored. Evolutionary strategies allow the optimization of a molecule's properties by a cyclic process consisting of consecutive variation and selection steps [13]. For this stepwise improvement of molecular parameters, no *a priori* knowledge of quantitative structure-activity relationships (QSAR) is required and the whole process may take place *in vitro*, *in vivo* or even *in silico* by computer-based algorithms.

The common QSAR approach consists of two main elements that could be considered as coding and learning [14]. The learning part can be solved with standard machine learning tools. Artificial neural networks are commonly used in this context as nonlinear regression models that correlate biological activities with physiochemical or structural properties. The coding part is based on identification of molecular descriptors that encode essential properties of the compounds under investigation [14]. Alternative approaches of classical machine-learning-based QSAR described above circumvent the problem of computing and selecting a representative set of molecular descriptors. Therefore molecules are considered as structured data–represented as graphs–wherein each atom is a node and each bond is an edge. These graphs define the topology of a learning machine. This is the main concept of the molecular graph network [15], the graph machines [16] and the graph neural network model [17] in chemistry which translate a chemical structure into a graph that works as a topology template for the connections of a neural network.

Artificial neural networks are computer programs inspired by nature that were intended to process complex information in a manner similar to the human brain [18], [19]. Although they did not fulfill the high expectations of the early days, they evolved into useful non-linear statistical modeling tools. In this role they have been successfully used in the QSAR field, generating hypotheses in the drug design cycle for GPCRs and other target classes and in automated feature extraction, yielding convincing results in numerous projects for small molecule drug development [19]–[25,25]. Artificial neural networks have also generated very substantial progress in the optimization of peptides for various purposes in molecular biology and pharmaceutical design, for instance in MHC I binding and stabilizing peptides [26]–[28], for the identification and biological activity of signal peptidase and viral proteinase cleavage sites [29]–[31], with cell-interacting peptides [32], and in modifying peptide transport across the blood-brain barrier [33]. Schneider et al. [34] have used artificial neural networks and a computer-based evolutionary search to design autoantibody-binding peptides by a cyclic variation-selection process. Using multiple iterations of synthesis, assay and computer-based molecular design, Riester et al. [35] were able to identify enhanced peptidic thrombin inhibitors. Their approach was based on an efficient type of genetic algorithm [36]. Currently, the optimization process for GPCR peptide ligands involves traditional and modern approaches in medicinal chemistry, as reviewed in [37]. So far, the optimization of GPCR peptide ligands by artificial neural networks has not been described.

In this work, we follow the idea of translating chemical structure directly into the topology of a learning machine. Our strategy is focused on peptides, wherein the sequence of amino acids determines the topology of the neural network. Each cell in the network corresponds one-to-one to an amino acid in the peptide. Learning the QSAR means adopting the weights of the cells in the network with respect to the quantity of interest (in general the activity or the metabolic stability). The architecture of this neural network fits into the concept of the Graph Neural Network [17]. In contrast to the Self Organizing Maps (also known as Kohonen maps [38]) they are trained using supervised learning and the topology is defined by the peptide sequence. The adapted cells are used to build models for the QSAR of new virtual peptides in order to optimize the desired property *in silico*. We explore the multidimensional space of all possible peptides with a genetic algorithm (GA) wherein the output of the GNN model defines the fitness function of the GA. Only the top ranking virtual peptides are selected for synthesis and in vitro testing in order to produce new measurements for the next optimization cycle.

Chemerin is a 163 amino acid polypeptide identified as a natural ligand for the heptahelical transmembrane receptor CMKLR1 [39]. Initially, the chemerin gene (also known as retinoic acid receptor responder 2 [RARRES2] or tazarotene-induced gene [TIG2]) had been identified as a novel retinoid-activated gene in skin [40]. It has been reported to be involved in the regulation of immune responses and adipogenesis, being engaged in a variety of physiological functions [41]–[47]. Its precursor molecule prochemerin is proteolytically activated and finally inactivated in sequential steps, modulating its physiological role in tissues [48]–[50]. Chemerin-9 (chemerin 148–156) was previously identified as a peptide mimic of full-length chemerin showing low nanomolar potency [51], [52]. Chemerin is also measurable in a number of human inflammatory exudates, including ascitic fluids from human ovarian cancer and liver cancer, as well as synovial fluids from arthritic patients. In the wake of chemerin's role as an adipokine and inflammatory modulator, a stabilized CMKLR1 ligand of high affinity may be beneficial in the treatment of metabolic syndrome and chronic inflammatory diseases.

By applying several cycles of peptide synthesis, testing in bioassays and GNN-based sequence optimization we have gradually improved the metabolic stability of a CMKLR1 nonamer peptide ligand with agonistic properties. For the first time, we describe the application of a novel GNN technology for the optimization of a small peptide for potential pharmaceutical application. By using this approach, we were able to achieve a 70fold improvement of the metabolic half life (t_½_ = 1693 min) in a ligand with subnanomolar activity in the biological assay (EC_50_ = 0.49 nM).

## Results

### Graph Neural Networks and Genetic Algorithms

**Figure 1:**
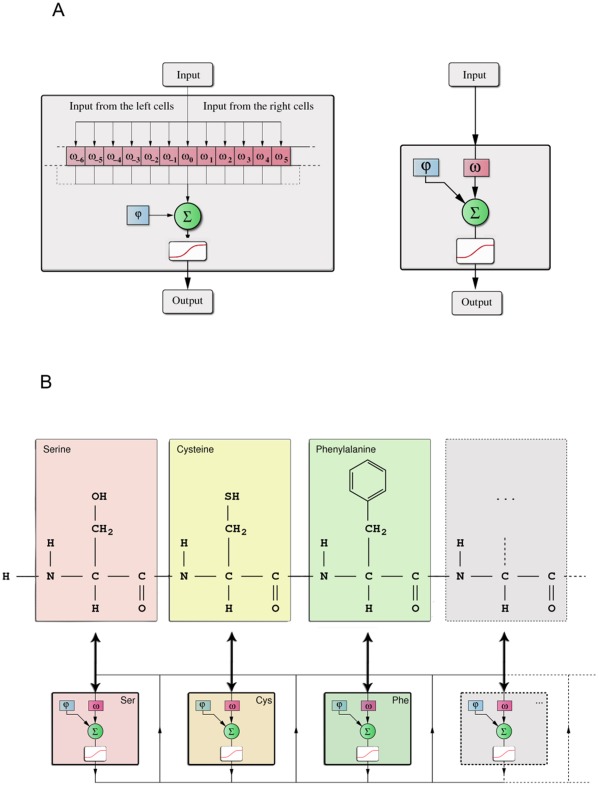
(A) Graph Neural Networks consist of interconnected elementary cells. The internal weight of the cell is given by φ_internal_ and the feedback during the iteration of the GNN is controlled by ω_0_. The inputs from the neighboring cells are connected with the weights ω_-N,…,N_ preserving the order of the peptide sequence as shown in (B). The sum of the weighted inputs passes the activation function and forms the output of the cell for the next iteration step of the network. (B) The translation process of a peptide into a fully connected GNN. Note the one-to-one correspondence between the amino acids of the peptide and the elementary cells of the GNN. The network is iterated through time and the output of the network is the sum over all internal states after the final iteration.

The GNN was designed to reflect the topology of a peptide by mimicking the sequence and the type of the constituting amino acids. Each amino acid had a representation as a particular elementary cell in the network with individual weights that were adjusted during the network training. Figure 1 shows a schematic plot of an elementary cell together with the weight vector that defines the connections to the neighboring cells of the network.

**Figure 2:**
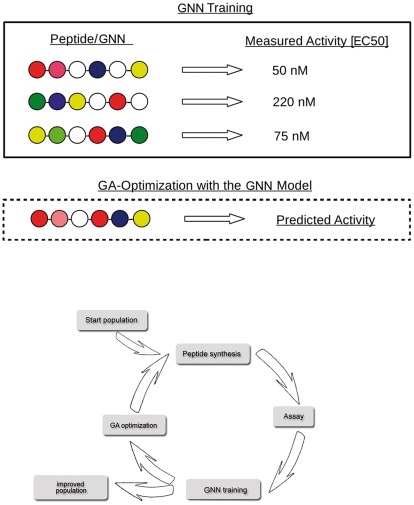
GNNs are trained and then optimized. This example illustrates the GNN's mode of action in computer-assisted peptide design: In the training set, the model is taught the properties of the current peptides (biological activity, stability) and the adopted cells build virtual peptides that are evaluated in the genetic algorithm-based optimization. The peptide optimization process is organized in multiple consecutive cycles. The start population of peptides is based on experts' knowledge concerning the target, e.g. natural ligands, known analogs or compounds that bind to similar targets. The trained GNN is used as a fitness function in a genetic algorithm. Newly generated sequences then have to be synthesized and analyzed in biological assays, before the next GNN training is initiated.

GNN training was initiated by feeding in the peptides' current sequences and biochemical properties such as agonistic activity and metabolic stability (Fig. 2). The training procedure was based on stochastic gradient descent with several improvements that make the training of the shared weights feasible [53]. The application of GNN-based molecular optimization was organized in a circular fashion (Fig. 2). From a start population of molecule sequences derived from established knowledge, peptides were synthesized and their properties were determined using the appropriate biochemical or cellular assay.

A GNN was then trained as described above and the adapted cells worked as building blocks of new virtual peptides that were generated by rearranging the order of the cells. The resulting GNN-model defined the fitness function in a GA that was generating an ensemble of improved peptide sequences entering the next cycle of development.

### Generation of an initial set of peptide variants (cycle 0)

In order to generate a diversity of structural variants in round 0 of the optimization process, 30 different sequences were derived from modifications of the native nonamer of chemerin-9. These variants were synthesized as peptide amides and subjected to EC_50_ determination for agonistic activity on HEK293 cells transfected with CMKLR1, the receptor for chemerin (start population or input, data not shown). In this initial round of screening, we explored the capacity for the exchange against other (natural) L-amino acids and stabilizing D-amino acids in the activation assay – no stability data were obtained at that stage. To allow better comparison with data from later cycles, the five peptides with the best EC_50_ values were resynthesized with a free acid function at the C terminus and were analyzed for their EC_50_ and metabolic stability (Table 1, cycle 0). As can be seen from the comparison with data from the native nonamer chemerin-9 (Table 1, bottom line), this initial round did not improve the mean stability of the peptide variants. Instead, half life of all these peptides in human serum was similar to the wild-type molecule (24 mins).

**Table 1:** Sequences and bioassay data of the five best peptides from each cycle.

optimization cycle	peptide sequence	activity: EC_50_ ±SD [nM]	stability: t_1/2_ ±SD [min]
0	YFPG**N**FAFS	9.96±4.42	16±2
0	Y**L**PGQFf**F**S	0.77±0.37	24±3
0	**Y**FPGQYAF**F**	1.89±0.90	26±8
0	YFPGQFAF**G**	0.79±0.54	13±1
0	YFPG**H**FAFS	6.63±2.91	17±4
1	**FL**PGQ**Y**AFS	2.75±0.14	28±1
1	Y**L**PGQ**Y**AF**L**	5.75±2.56	27±4
1	Y**L**PGQFAF**s**	1.72±0.32	31±0
1	Y**V**PGQFAF**f**	4.17±2.31	42±19
1	**yL**PGQ**Y**AF**F**	9.23±3.68	135±39
2	**fL**PGQ**Y**AFf	8.20±3.96	1157±41
2	Y**L**PGQ**Y**AF**f**	0.21±0.22	36±4
2	Y**L**PGQYAF**s**	0.40±0.23	22±2
2	**yL**PGQ**Y**AFS	3.62±2.91	77±5
2	Y**R**PGQ**Y**AF**s**	8.62±5.01	21±4
3	Y**L**PaQ**Y**AF**s**	4.42±3.41	110±26
3	Y**L**PGQ**Yw**F**f**	3.24±1.02	159±56
3	Y**L**Pq**Q**YAF**f**	4.24±1.87	87±19
3	**yL**P**s**Q**Y**AF**f**	**0.49**±0.13	**169**±291
3	**yL**P**s**Q**Y**AF**s**	2.90±1.34	1408±85
chemerin-9	YFPGQFAFS	**1.29**±1.54	**24**±6

Peptides from the initial population are designated as from cycle 0. In the peptide sequences, wild type amino acids are given in standard font. Exchange by a different L amino acid is represented by a character in bold, D isomers are in addition indicated by lower-case letters. Activities are intracellular calcium mobilization data given as mean of three independent experiments ± standard deviation. Stabilities are peptide half life data from an HPLC assay, given as mean of three independent experiments ± standard deviation. Data in bold indicate the best peptide in this study, showing approximately 70fold higher stability and 2.5fold higher potency than the wild-type peptide chemerin-9.

### GNN improvement (cycle 1–3)

**Figure 3:**
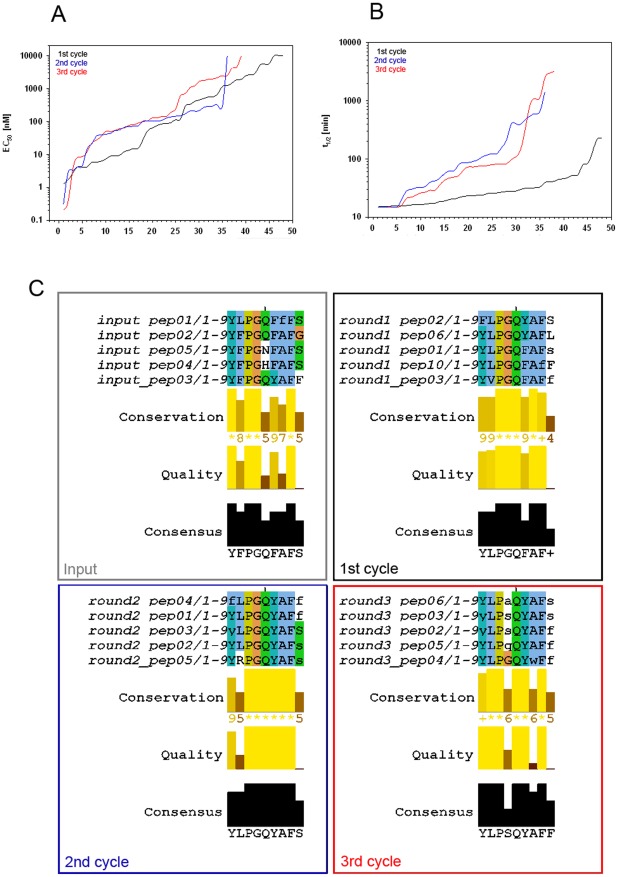
As peptide sequences evolve, metabolic stability of the peptides improves while their biological activity is retained. Upper panel (A+B): depiction of EC_50_ values of the receptor activating potency (A) and of the metabolic stability (B,  = t_1/2_) for the three GNN-based optimization rounds. Both graphs show all the peptides from a given round, sorted according to their activity in the parameter on the y axis. With receptor activity, low EC_50_ values are favorable, while in the stability parameter t_1/2_, high values are to be achieved. Lower panel (C): Peptide sequence comparisons by multiple alignments illustrate the evolution over the different steps of the process. Four sets of alignments represent the start population ( = input) and three optimization rounds.

Round 1 to 3 of the optimization process included at least 34 different sequences each from a GNN model as described above (Table S1). These variants were synthesized as peptides with a free acid function at their carboxy terminus and were subjected to EC_50_ determination for agonistic activity on HEK293 cells expressing CMKLR1. In these subsequent rounds of screening, we explored the capacity for the exchange against other (natural) L-amino acids and also stabilizing D-amino acids in the activation assay, as well as the metabolic stability of the various compounds. As can be seen from the comparison with data from the native nonamer chemerin-9 (Table 1, bottom line), and with results from round 0, the following rounds of optimization yielded a marked improvement in metabolic stability while retaining high agonistic activity. Quite obvious is the gradual enhancement over the three rounds: both bioactivity (EC_50_) and half-life of the peptides improve from first to third cycle (Fig. 3 A and B).

### Analysis of the five best peptides from each cycle

To analyze the structure-activity relationships in the process of GNN optimization, multiple sequence alignments of the five best peptides from each cycle were compared (Fig. 3C). The sequences from **cycle 0** (input or start population) showed moderate conservation across the peptide, with higher variability towards the carboxyterminal portion. Here, at the beginning of the evolutionary process, the consensus sequence of these five peptides still corresponded to the chemerin-9 wild type sequence (Fig. 3C, upper left graph). The aromatic amino acids at positions 1, 2 and 8 as well as the sterically peculiar Pro^3^ and Gly^4^ were well conserved. The only exception was found in a single exchange of Phe^2^ by Leucin. The same peptide also showed the only inclusion of a D amino acid (D-Phe^7^) which, however, did not lead to any improvement in stability over the wild type (Table 1, 2^nd^ and last line). Position 9 is Serin in the wild type, but it obviously easily accommodates exchanges with either aromatic or small hydrophobic amino acids (Phe or Gly).

Within the five best sequences of **cycle 1** (Fig. 3A, upper right graph), the Leu^2^ introduction from cycle 1 was further inherited, dominating this position with only one exception (Val^2^). Again, Pro^3^ and Gly^4^ were unchanged in addition to the aromatic position 1, Glu^5^ and Phe^8^. Both these trends lead to a consensus sequence close to wild type, with only position 2 substituted by Leu, while position 9 was variable. In this set of peptides, the first significant improvement in stability is associated with the *de novo* introduction of a D amino acid at the amino terminal position 1 (Table 1, 10^th^ and last line), increasing the half life by a factor of 5. Two peptides with a D amino acid at the carboxy terminus did not show prolonged stability.

In **cycle 2** of the GNN optimization, however, the algorithm continued to introduce carboxyterminal D amino acids and to a lesser extend also at the amino terminus, while no internal positions of the five best peptides in this round contain the D isoform. Here, the combination of D amino acids at both termini yields the first peptide with considerably higher stability, increasing the half life by a factor of 48 to 1157 minutes (Table 1). Positions 3 to 8 were completely conserved in this set of peptides, including Tyr^6^, first introduced in cycle 1. The consensus sequence consequently differs in positions 2 and 6 from the wild type (Fig. 3A, lower left graph).

**Cycle 3,** as the previous cycle, yielded a novel exchange at a previously conserved position, leading to two peptides with additional improvement of stability as well as enhanced activity: Gly^4^ is substituted by D amino acids. Of these substitutions, D-Ser proved to be most effective, leading to stabilities of 1408 and 1693 minutes (59fold and 70fold improvement, respectively). At the same time, D-Ser lead to a peptide with subnanomolar activity at the chemerin receptor (0.49 nM, data highlighted in bold in Table 1). In this cycle, the five best peptides showed high conservation at all other positions, with some variability towards the carboxy terminus. The consensus sequence in this last round differed in four positions (2, 4, 6, 9) from the original chemerin-9, the exchange of Gly by D-Ser at position 4 being the most prominent one (Table 1 and Figure 3A, lower right graph).

**Figure 4:**
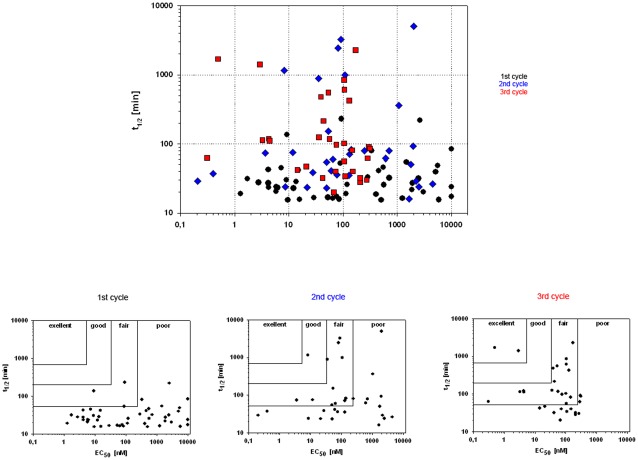
**Stability and activity of chemerin peptides improve over three cycles. Upper panel:** The complete data set from three optimization rounds is shown, each dot representing one peptide and its properties. The trend points to the upper left part of the diagram, i.e. peptides that combine high receptor activity ( = low EC_50_) with high metabolic stability. **Lower panel:** separate representations of the data from all three rounds. While in the first round, the majority of data points is found in the lower right quadrant with poor stability and activity, in cycles 2 and 3 the data cloud is shifted towards the upper left, representing improved activity and stability. Quality is a measure of the likelihood of observing the substitutions in a particular column of the alignment [62].

The effectiveness of the GNN-based modulation of the peptide composition are visualized in plots for both parameters analyzed within this process (Fig. 4). While in the first round, the majority of data points were found in the lower right quadrant with poor stability and activity, in cycles 2 and 3 the data cloud was shifted towards the upper left, representing improved activity and stability.

## Discussion

We presented a Graph Neural Network (GNN) that utilizes individual processing elements as building blocks with a one-to-one correspondence to the amino acids of the peptide. The GNN mimics the linear design of a peptide molecule and converts this chemical architecture directly into the topology of a learning machine. This strategy eliminates the obstacle of designing and computing molecular descriptors for QSAR. In addition, the GNN requires no preexisting structural knowledge about the drug target. The scarcity of available 3D structural information on GPCRs and other membrane-bound proteins thus does not limit the GNN concept. The novelty of our approach lies in the topology preserving network structure that mimics the peptide chain and in that the learning takes place in the weights of the cells that correspond to the amino acids of the peptide. With the adapted cells, we are able to assemble new *virtual* peptides and the resulting GNNs define the fitness function in a genetic algorithm that is used to search for new peptides.

The feasibility of this approach was demonstrated in the construction and optimization of CMKLR1 ligands in an iterative process of three design cycles of computer-assisted optimization with respect to the biological activity and metabolic stability of the peptides. Every round of optimization included synthesis of candidate molecules, characterization of these peptides in bioactivity and stability assay, and algorithmic processing of the data to generate or improve the GNN that links compound structure and molecular properties. This in turn generates a new set of peptide sequences. Starting from an initial set of randomly chosen chemerin-9 variants, a multiparameter optimization was carried out in three molecular design cycles. We investigated 9-mer peptides from an alphabet consisting of the 20 natural proteinogenic amino acids and 15 D-amino acids. Synthesis and experimental fitness determination of less than 160 different compounds from the resulting virtual combinatorial library of more than 7.8×10^13^ peptide nonamers were necessary to achieve this goal. Our GNN strategy together with the GA-based exploration of the combinatorial peptide space is the core concept of a novel peptide optimization process in drug discovery. It allows one to efficiently screen the huge chemical space generated by the combinatorial explosion of possible virtual peptide sequences. For the first time, artificial neural networks were used to substantially improve the properties of a peptide receptor ligand.

Various types of artificial neural networks have been utilized to enhance structure-function optimization with many different molecular entities [25], [54]. Schneider et al. [34] have pioneered the artificial neural network design of peptides, identifying novel sequences that block anti-β1-adrenoreceptor autoantibodies. Starting from a single seed peptide, these authors trained their neural networks on sequence-activity relationships to generate *de novo* peptide sequences with considerable biological activity. In contrast, the peptides with the most favorable properties from our study still retain a common sequence pattern with wild-type chemerin-9; P^3^, Q^5^, A^7^, F^8^ are present in the two best peptides. In addition, Y^1^ is changed to a D-amino acid and F^2^ and F^6^ are conservatively exchanged for L or Y. Even among peptides with lower stability but retained high activity, few variations from the native YFPGQFAFS sequence of chemerin-9 occur.

Riester et al. (21) organize their optimization process in design cycles starting with a randomly chosen set of molecules. They run a GA on the sequence level that has been tailored to meet the demands of *de novo* drug design (small training sets, fast convergence, and few design cycles). In contrast to this approach, we run a GA on the building blocks of a GNN model that was designed to cover possible interactions between distant amino acids. In this way, the GNN approach is much more adaptive than a static sequence based GA because it translates chemical structure directly into the topology of a learning machine. The past years have seen a steady increase of reports on the role of chemerin in a variety of physiological processes as well as in disease conditions, ranging from inflammation to diabetes, obesity and hypertension (for a review, see [55]). Still, the exact role of chemerin in these disorders remains to be elucidated and further research is required to determine the significance of high serum levels in corresponding patients. However, new data on disease pathology will potentially unravel novel therapeutic approaches that may involve stable analogs derived from the short active peptide chemerin-9.

Shimamura et al. [56] have recently reported a different approach to generate stabilized chemerin-9 variants. They substituted amino acid positions at predicted protease cleavage sites and analyzed the effect of these modifications by HPLC and mass spectrometry. Using the same readouts as in this study (Ca^2+^ mobilization and serum stability assay), the authors were able to create a chemerin-9 variant with a reported half-life of >240 min. The peptide contains one non-natural building block and three more D-amino acids and has an EC_50_ in the Ca^2+^ mobilization assay of 22 nM. While the peptide from our own study showed a half-life of nearly 1700 minutes, the utility of this molecule for in vivo use still needs to be demonstrated by an in vivo pharmacokinetic study. However, the prolongation of the peptides' metabolic stability in serum by a factor of 70 appears to be a very promising starting point for the translation of this molecule into a therapeutic entity.

## Materials and Methods

### Neural Network Designs

The building blocks of a GNN are the elementary cells that are in silico representations of the amino acids in the peptide. An elementary cell has a weight vector containing an internal weight and weights that control the interactions with neighboring cells. The elementary cells are connected to form a chain with one-to-one correspondence between the amino acids that build the peptide and the cells in the network. The internal weight of the cell is φ, the inputs from the neighboring cells are connected with the weights 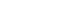 and the feedback is controlled by 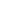. The weights are combined in the weight vector 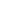. The translation of a peptide sequence into a GNN is based on the one-to-one correspondence between the amino acids of the peptide and the elementary cells of the GNN. The GNN is iterated several times which governs the system dynamics. The number of iterations **T** is set to be the average length of the sequences under investigation. The state of the i-th GNN cell 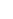 evolves for iterations t = 0,…, **T**-1 according to:
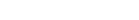





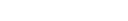
.

The output of the GNN is the average of the internal states after T iterations.

### Training GNN Models

Training a GNN is put to effect by minimizing the training error with respect to the network weights based on stochastic gradient descent. The true gradient is approximated by the gradient of the loss function which is evaluated on single training samples. The network weights are adjusted by an amount proportional to this approximate gradient. A training sample consists of two parts: The first part is the peptide sequence that is a composition of the **M** possible amino acids taken from the alphabet. The second part is the measured activity that could be a continuous value or a class label. We assume to have a collection of **K** training samples and the weight vectors of the individual cells in the network that correspond to the **M** different amino acids in the alphabet are organized in the weight vector . Let denote the output of the GNN for a given sequence with respect to the network weights . This output value has to be compared to the training label by means of a loss function . The loss function measures the deviation of the network output from the desired value . The training error is simply the loss averaged over the entire training set.

.

Training the network means minimizing the training error with respect to the network weights based on stochastic gradient descend. The stochastic gradient descent performs a series of very small consecutive steps, determining the direction of each step from the gradient of an individual error term of the form

.

After each step, the new weights Ω are re–inserted into the loss function before the next gradient is computed. This defines an update rule for the weights of the form:

with i = 1,…, K wherein K is the number of training samples. The update ΔΩ_i_ depends on the i-th training sample only and is given by

We calculate the update ΔΩ_i_ with the standard error back-propagation technique as it is used for the common feed-forward multilayer neural network. Details of the training procedure are described in Wichard et al. [39]. The parameter µ controls the step size of the gradient descend. The initial step size is already small (around µ = 0.01) and it is decreased after each training epoch with a constant factor. This is necessary to achieve a slow convergence of the weights. Note that in each training step only a few selected values of the entire weight vector Ω are adjusted, namely the ones that correspond to amino acids that appear in the sequence of the training sample.

In order to build models with good generalization abilities we applied two common techniques: Normalization and ensemble building. For normalization we added a small weight decay term to the loss function that penalizes large weights in the network and causes the insignificant weights to converge to zero [57].

Ensemble building is a well established method that can improve model generalization. In general, neural network ensembles perform better in terms of generalization than single models would do [58], [58,59]. For ensemble building, we followed a cross-validation strategy: An ensemble of GNNs consists of several single models that are trained on randomly chosen subsets of the data with random weight initializations. This ensures the diversity of the resulting models which is the key issue in ensemble building. The validation data consists of data points that were hold out from the training. Only GNN models that perform well on the validation data are used for the final ensemble.To compute the ensemble-output for one input sequence, the output variables of all GNN belonging to the ensemble are averaged. More details of the GNN training procedure were described in [53].

### GNN Models as Fitness Function in a Genetic Algorithm

The main objective in building a GNN model is to recover the fundamental characteristics of the structure activity relation. The adapted cells of a fully trained GNN model work as building blocks of new peptides which are generated by rearranging the order of the cells and calculating the output of the network. This defines the fitness function in a genetic algorithm (GA) that is generating new suggestions for peptide synthesis based on the learned structure activity relation. The GA performs adaptation by identifying and recombining schemata, i.e. substrings with above average fitness, following the building block theory introduced by Holland [60]. We perform mutation and 2-point crossover on the sequence level. The start population consists of 2000 randomly generated peptide sequences and evolves over 5000 generations with ‘elitist selection’, i.e. keeping the best performing individuals of the population unchanged.

### Peptide synthesis and analytics

All peptides were synthesized by peptides&elephants GmbH (Potsdam, Germany) in 2 mmol scale on a LIPS® 96 peptide synthesizer. Synthesis was carried out in resin-preloaded MultiPep 96® microtiter plates (peptides&elephants GmbH, Potsdam, Germany) using Fmoc chemistry on Rink amide AM resin or N-biotinyl-NFmoc-ethylenediamine-MPB AM resin (Merck Biosciences AG, Darmstadt, Germany). All solvents were of reagent or HPLC grade and were bought from Carl Roth GmbH (Karlsruhe, Germany). Temporary Fmoc protection groups were removed by treatment with 20% piperidine v/v in dimethyl formamide. Amino acid coupling was done with 4 equiv activated amino acid solution (0.2 M in N-methyl pyrrolidone). Permanent protection groups were removed and peptides were released from the resin by treatment with 90% TFA, 5% triisopropyl silane, 2.5% DTT, and 2.5% HPLC-water v/v. Peptides were lyophilized, redissolved in TFA, and precipitated by the addition of ice-cold hexane-diethylether solution (50/50). All peptides were analyzed by MS (Finnigan Surveyor MSQ Plus, Thermo Finnigan, Bremen, Germany) to confirm the presence of the correct molecular mass. For each library synthesized, at least 10% of the peptides were analyzed for purity using HPLC. Mean purities of the nonamer synthesis raw products used in the assays ranged from 78 to 93%.

### Cell Culture and Transfection

HEK293A-cells (Invitrogen) were grown in RPMI 1640 medium buffered with 2.0 g/l NaHCO_3_ (Biochrom AG) supplemented with 10% FCS, 2 mM L-glutamine, penicillin-streptomycin (100 IU/mL; 100 µg/mL) at 37°C in a humidified 5% CO2 incubator. Cells were split into poly-D-lysine coated black-wall 96-well black well/clear bottom plate (BD Falcon) at a density of 5*10^4^ cells/well and cultured for an additional period of 18- to 24-h. Afterwards, cells stable expressing human G_α15_ were transfected with human CMKLR1 pcDNA3.1 (Missouri S&T cDNA Resource Center) construct using the jetPEI (peqlab) transfection reagent according to the supplier's protocol. Two days after transfection, cells were selected in RPMI 1640 medium buffered with 2.0 g/L NaHCO_3_ (Biochrom AG), supplemented with 10% fetal calf serum, 2 mM L-glutamine, penicillin-streptomycin (100 IU/mL–100 µg/mL), 150 µg/mL zeocin and 300 µg/mL G418 for a period of 4 weeks (37°C, 5% CO_2_, 95% relative humidity). Functional expression of human G_α15_ and CMKLR1 in cells was verified in a CellLux assay recording CMKLR1 / G_α15_ mediated intracellular Ca^2+^ release upon receptor activation with chemerin-9, as described below.

### Ca^++^ Imaging

HEK293A cells (Invitrogen) expressing CMKLR1 and Gα15 were seeded at 5*10^4^ cells/well in a poly-D-lysine coated 96-well black well/clear bottom plate (BD Falcon) and cultured overnight. From eighteen to twenty-four hours later, cells were loaded with FLUO4-AM (Invitrogen). Then the cells were washed two times with C1 solution (130 mM NaCl, 5 mM KCl, 10 mM HEPES pH 7.4, 2 mM CaCl_2_, and 10 mM Glucose). After the final wash, a 100 µL of residual volume remained on the cells. Peptides were dissolved in 10% DMSO to a concentration of 1 mM and were diluted in C1 solution with 0.1 % BSA. They were aliquoted as 2x solutions in 96-well plates and were simultaneously transferred by the robotic system within the imager from the ligand plate to the cell plate. Fluorescence was recorded simultaneously in all wells using an imaging plate reader CellLux (PerkinElmer) at an excitation wavelength of 488 nm and emission wavelength of 510 nm at 1.5 s intervals over a period of 4 min. Fluorescence data was generated in duplicate and experiments were repeated for at least three times. We tested all compounds at eleven different concentrations over a range of 5 orders of magnitude. For the calculation of concentration-response curves, signals of two wells receiving the same concentration of test substances were averaged and the fluorescence changes of corresponding buffer C1 wells were subtracted. Signals were normalized to background fluorescence. For the calculation of EC_50_ values, plots of amplitude versus concentrations were prepared in SigmaPlot 11. By nonlinear regression of the plots we were able to calculate the EC_50_ of agonist-receptor interaction.

### In Vitro Peptide Stability in Serum/Reaction Kinetics

In vitro peptide stability assays were carried out as previously described from [61]. Briefly, 500 µL of RPMI supplemented with 25% (v/v) of human serum are allocated into a 1.5 mL Eppendorf tube and temperature-equilibrated at 37 ±1°C for 15 min before adding peptide stock solution to make a final peptide concentration of 50 µg/mL. Dilution of the serum will result in the proteolytic enzymes being the limiting factor and enable a linear degradation of the peptides over time. The initial time is recorded, and at known time intervals 50 µL of the reaction solution is removed and added to 100 µL of 6% aqueous trichloroacetic acid (TCA) for precipitation of serum proteins. The cloudy reaction sample is cooled on ice for 15 min and then spun at 18,000 ***g*** (Eppendorf centrifuge) for 2 min to pellet the precipitated serum proteins. The reaction supernatant is then analyzed using RP-HPLC (Agilent 1200 LC System) on a ZORBAX Eclipse XDB-C18, 4.6×150 mm, 5 µm column (Agilent). A linear gradient from 25% to 80% acetonitrile, is used over 15 min with a flow rate of 1 mL/min at 30°C. Absorbance is detected at 214 nm and 280 nm. Fluorescence is detected at an excitation wavelength of 280 nm and emission wavelength of 340 nm. Kinetic analysis was carried out by least-squares analysis of the logarithm of the integration peak area versus time.

### Sequence analysis

Multiple alignments of peptide sequences and their graphic representation were generated using the *Jalview* software package V2.6.1 [62].

## Supporting Information

Table S1 This table provides a complete list of all peptides used in this study along with their sequence and bioassay data. All peptides in the start population were amidated variants (indicated by -NH2 at the end of the sequence). The table also states the biological activity or potency in the Ca++ mobilizations assay (EC_50_), the maximum response in this assay, and the biological half life in human serum (t_½_). These data all result from at least three independent experiments done in duplicate, variation is given as standard deviation (SD).
(DOC)

## References

[1] Marinissen MJ, Gutkind JS (2001). G-protein-coupled receptors and signaling networks: emerging paradigms..

[2] Lagerstrom MC, Schioth HB (2008). Structural diversity of G protein-coupled receptors and significance for drug discovery.. Nature Reviews Drug Discovery.

[3] Lappano R, Maggiolini M (2011). G protein-coupled receptors: novel targets for drug discovery in cancer.. Nature Reviews Drug Discovery.

[4] Jacoby E, Bouhelal R, Gerspacher M, Seuwen K (2006). The 7TM G-protein-coupled receptor target family.. Chemmedchem.

[5] Palczewski K, Kumasaka T, Hori T, Behnke CA, Motoshima H (2000). Crystal structure of rhodopsin: A G protein-coupled receptor.. Science.

[6] Murakami M, Kouyama T (2008). Crystal structure of squid rhodopsin.. Nature.

[7] Rasmussen SGF, Choi HJ, Rosenbaum DM, Kobilka TS, Thian FS (2007). Crystal structure of the human beta(2) adrenergic G-protein-coupled receptor.. Nature.

[8] Warne T, Serrano-Vega MJ, Baker JG, Moukhametzianov R, Edwards PC (2008). Structure of a beta(1)-adrenergic G-protein-coupled receptor.. Nature.

[9] Jaakola VP, Griffith MT, Hanson MA, Cherezov V, Chien EYT (2008). The 2.6 Angstrom Crystal Structure of a Human A(2A) Adenosine Receptor Bound to an Antagonist.. Science.

[10] Wu B, Chien EY, Mol CD, Fenalti G, Liu W (2010). Structures of the CXCR4 chemokine GPCR with small-molecule and cyclic peptide antagonists..

[11] Chien EYT, Liu W, Zhao QA, Katritch V, Han GW, Hanson MA, Shi L, Newman AH (2010). Structure of the Human Dopamine D3 Receptor in Complex with a D2/D3 Selective Antagonist.. Science.

[12] Shimamura T, Shiroishi M, Weyand S, Tsujimoto H, Winter G (2011). Structure of the human histamine H(1) receptor complex with doxepin.. Nature.

[13] Willett P (1995). Genetic algorithms in molecular recognition and design.. Trends in Biotechnology.

[14] Terfloth L, Gasteiger J (2001). Neural networks and genetic algorithms in drug design.. Drug Discovery Today.

[15] Merkwirth C, Lengauer T (2005). Automatic generation of complementary descriptors with molecular graph networks.. Journal of Chemical Information and Modeling.

[16] Goulon A, Duprat A, Dreyfus G (2006). Graph machines and their applications to computer-aided drug design: A new approach to learning from structured data.. Unconventional Computation, Proceedings.

[17] Scarselli F, Chung Tsoi A, Hagenbuchner M, Monfardini G (2009). The Graph Neural Network Model.. IEEE Transactions on Neural Networks.

[18] Agatonovic-Kustrin S, Beresford R (2000). Basic concepts of artificial neural network (ANN) modeling and its application in pharmaceutical research..

[19] Krogh A (2008). What are artificial neural networks?.

[20] Zupan J, Gasteiger J (1993). Neural Networks for Chemists: An Introduction.

[21] Devillers J (1996). Neural Networks in QSAR and Drug Design.

[22] Tetko IV, Tanchuk VY, Chentsova NP, Antonenko SV, Poda GI (1994). HIV-1 reverse transcriptase inhibitor design using artificial neural networks.. J Med Chem.

[23] Lopez-Rodriguez ML, Morcillo MJ, Fernandez E, Rosado ML, Pardo L, analysis Studyofthe5-HT(1a)/alpha(1)-adrenergicreceptoraffinitybyclassicalhansch, networks artificialneural (2001). Synthesis and structure-activity relationships of a new model of arylpiperazines.. and computational simulation of ligand recognition.

[24] Balakin KV, Lang SA, Skorenko AV, Tkachenko SE, Ivashchenko AA (2003). Structure-based versus property-based approaches in the design of G-protein-coupled receptor-targeted libraries..

[25] Schneider G, Wrede P (1998). Artificial neural networks for computer-based molecular design..

[26] Milik M, Sauer D, Brunmark AP, Yuan L, Vitiello A, Jackson MR, Peterson PA, Skolnick J, Glass CA (1998). Application of an artificial neural network to predict specific class I MHC binding peptide sequences..

[27] Hiss JA, Bredenbeck A, Losch FO, Wrede P, Walden P (2007). Design of MHC I stabilizing peptides by agent-based exploration of sequence space..

[28] Wisniewska JM, Jager N, Freier A, Losch FO, Wiesmuller KH (2010). MHC I stabilizing potential of computer-designed octapeptides..

[29] Jagla B, Schuchhardt J (2000). Adaptive encoding neural networks for the recognition of human signal peptide cleavage sites.. Bioinformatics.

[30] Wrede P, Landt O, Klages S, Fatemi A, Hahn U (1998). Peptide design aided by neural networks: biological activity of artificial signal peptidase I cleavage sites..

[31] Du QS, Huang RB, Wei YT, Wang CH, Chou KC (2007). Peptide reagent design based on physical and chemical properties of amino acid residues..

[32] Kaga C, Okochi M, Tomita Y, Kato R, Honda H (2008). Computationally assisted screening and design of cell-interactive peptides by a cell-based assay using peptide arrays and a fuzzy neural network algorithm..

[33] Teixido M, Belda I, Zurita E, Llora X, Fabre M, Design Part2 (2005). Evolutionary combinatorial chemistry, a novel tool for SAR studies on peptide transport across the blood-brain barrier.. synthesis and evaluation of a first generation of peptides.

[34] Schneider G, Schrodl W, Wallukat G, Muller J, Nissen E (1998). Peptide design by artificial neural networks and computer-based evolutionary search.. Proc Natl Acad Sci U S A.

[35] Riester D, Wirsching F, Salinas G, Keller M, Gebinoga M (2005). Thrombin inhibitors identified by computer-assisted multiparameter design..

[36] Kamphausen S, Holtge N, Wirsching F, Morys-Wortmann C, Riester D (2002). Genetic algorithm for the design of molecules with desired properties.. J Comput Aided Mol Des.

[37] Hruby VJ (2002). Designing peptide receptor agonists and antagonists..

[38] Kohonen T (1982). Self-Organized Formation of Topologically Correct Feature Maps.. Biological Cybernetics.

[39] Meder W, Wendland M, Busmann A, Kutzleb C, Spodsberg N (2003). Characterization of human circulating TIG2 as a ligand for the orphan receptor ChemR23..

[40] Nagpal S, Patel S, Jacobe H, DiSepio D, Ghosn C (1997). Tazarotene-induced gene 2 (TIG2), a novel retinoid-responsive gene in skin.. Journal of Investigative Dermatology.

[41] Bozaoglu K, Bolton K, McMillan J, Zimmet P, Jowett J (2007). Chemerin is a novel adipokine associated with obesity and metabolic syndrome..

[42] Cash JL, Christian AR, Greaves DR (2010). Chemerin peptides promote phagocytosis in a ChemR23- and Syk-dependent manner..

[43] Ernst MC, Issa M, Goralski KB, Sinal CJ (2010). Chemerin exacerbates glucose intolerance in mouse models of obesity and diabetes..

[44] Goralski KB, McCarthy TC, Hanniman EA, Zabel BA, Butcher EC (2007). Chemerin, a novel adipokine that regulates adipogenesis and adipocyte metabolism..

[45] Hart R, Greaves DR (2010). Chemerin contributes to inflammation by promoting macrophage adhesion to VCAM-1 and fibronectin through clustering of VLA-4 and VLA-5..

[46] Parlee SD, Ernst MC, Muruganandan S, Sinal CJ, Goralski KB (2010). Serum chemerin levels vary with time of day and are modified by obesity and tumor necrosis factor-{alpha}..

[47] Martensson UE, Fenyo EM, Olde B, Owman C (2006). Characterization of the human chemerin receptor–ChemR23/CMKLR1–as co-receptor for human and simian immunodeficiency virus infection, and identification of virus-binding receptor domains..

[48] Du XY, Leung LL (2009). Proteolytic regulatory mechanism of chemerin bioactivity.. Acta Biochim Biophys Sin (Shanghai).

[49] Du XY, Zabel BA, Myles T, Allen SJ, Handel TM (2009). Regulation of chemerin bioactivity by plasma carboxypeptidase N, carboxypeptidase B (activated thrombin-activable fibrinolysis inhibitor), and platelets..

[50] John H, Hierer J, Haas O, Forssmann WG (2007). Quantification of angiotensin-converting-enzyme-mediated degradation of human chemerin 145–154 in plasma by matrix-assisted laser desorption/ionization-time-of-flight mass spectrometry..

[51] Cash JL, Hart R, Russ A, Dixon JP, Colledge WH (2008). Synthetic chemerin-derived peptides suppress inflammation through ChemR23..

[52] Wittamer V, Gregoire F, Robberecht P, Vassart G, Communi D (2004). The C-terminal nonapeptide of mature chemerin activates the chemerin receptor with low nanomolar potency..

[53] Wichard J, Bandholtz S, Grötzinger C, Kühne R, Rutkowski-L (2010). Computer assisted peptide design and optimization with topology preserving neural networks.. ICAISC 2010.

[54] Melville JL, Burke EK, Hirst JD (2009). Machine learning in virtual screening.. Comb Chem High Throughput Screen.

[55] Ernst MC, Sinal CJ (2010). Chemerin: at the crossroads of inflammation and obesity..

[56] Shimamura K, Matsuda M, Miyamoto Y, Yoshimoto R, Seo T (2009). Identification of a stable chemerin analog with potent activity toward ChemR23..

[57] Lecun Y, Bottou L, Orr GB, Muller KR (1998). Efficient backprop.. Neural Networks: Tricks of the Trade.

[58] Perrone MPCLN (1993). When networks disagree: Ensemble methods for hybrid neural networks.. Chapman-Hall.

[59] Geman S, Bienenstock E, Doursat R (1992). Neural Networks and the Bias Variance Dilemma.. Neural Computation.

[60] Holland, H J (1975). Adaptation in natural and artificial systems..

[61] Jenssen H, Aspmo SI (2008). Serum stability of peptides..

[62] Waterhouse AM, Procter JB, Martin DM, Clamp M, Barton GJ (2009). Jalview Version 2–a multiple sequence alignment editor and analysis workbench..

