# Supplementary material for: Molecular Evolution of a Peptide GPCR Ligand Driven by Artificial Neural Networks
Source: PLoS One. 2012 May 14;7(5):e36948. doi: 10.1371/journal.pone.0036948 (PMC3351444; doi:10.1371/journal.pone.0036948)
Supplement: Table S1 — This table provides a complete list of all peptides used in this study along with their sequence and bioassay data. All peptides in the start population were amidated variants (indicated by -NH2 at the end of the sequence). The table also states the biological activity or potency in the Ca++ mobilizations assay (EC50), the maximum response in this assay, and the biological half life in human serum (t½). These data all result from at least three independent experiments done in duplicate, variation is given as standard deviation (SD). (DOC) [file pone.0036948.s001.doc]

**Supporting Information**

**Table S1**

| **optimization round** | **peptide** | **sequence** | **EC_50_ [nM]** | | | **max response [DF/F_0_]** | | | **t_1/2_ [min]** | | |
| --- | --- | --- | --- | --- | --- | --- | --- | --- | --- | --- | --- |
| start population | 1 | YFPGQYAFF-NH2 | 15,20 | ± | 10,14 | 1,47 | ± | 0,05 |  | ND |  |
| start population | 2 | YFPGQFAFK-NH2 | 37,26 | ± | 13,66 | 1,84 | ± | 0,11 |  | ND |  |
| start population | 3 | FLPGQFAFS-NH2 | 41,29 | ± | 28,07 | 1,77 | ± | 0,28 |  | ND |  |
| start population | 4 | YLPGQFfFS-NH2 | 44,73 | ± | 27,55 | 1,66 | ± | 0,30 |  | ND |  |
| start population | 5 | YFPGQFAFS-NH2 | 191,98 | ± | 73,00 | 1,46 | ± | 0,14 |  | ND |  |
| start population | 6 | YFPGNFAFS-NH2 | 214,08 | ± | 189,18 | 1,30 | ± | 0,49 |  | ND |  |
| start population | 7 | YFPGHFAFS-NH2 | 446,34 | ± | 154,13 | 1,55 | ± | 0,02 |  | ND |  |
| start population | 8 | yyPGQfAys-NH2 | 1878,45 | ± | 1155,99 | 1,31 | ± | 0,14 |  | ND |  |
| start population | 9 | YFPGQWAWS-NH2 | 1878,74 | ± | 756,32 | 1,61 | ± | 0,14 |  | ND |  |
| start population | 10 | YFPGIFAfS-NH2 | 2218,13 | ± | 687,87 | 1,48 | ± | 0,15 |  | ND |  |
| start population | 11 | YFPGYFAFG-NH2 | 2619,30 | ± | 1539,45 | 1,41 | ± | 0,13 |  | ND |  |
| start population | 12 | fFPGQWGFS-NH2 | 3012,52 | ± | 1052,22 | 1,56 | ± | 0,11 |  | ND |  |
| start population | 13 | YFPGQfAWS-NH2 | 3199,01 | ± | 2352,43 | 1,22 | ± | 0,13 |  | ND |  |
| start population | 14 | YFGpQFAFS-NH2 | 3444,10 | ± | 1963,41 | 1,34 | ± | 0,11 |  | ND |  |
| start population | 15 | ffPGQFGyn-NH2 | 3474,01 | ± | 1320,99 | 1,47 | ± | 0,13 |  | ND |  |
| start population | 16 | WYPGNFAFS-NH2 | 3840,15 | ± | 1272,93 | 1,47 | ± | 0,19 |  | ND |  |
| start population | 17 | YYPGQYAYS-NH2 | 4364,62 | ± | 1867,84 | 1,09 | ± | 0,11 |  | ND |  |
| start population | 18 | wFPGQFiFS-NH2 | 7128,22 | ± | 2569,03 | 1,40 | ± | 0,13 |  | ND |  |
| start population | 19 | YyPGqYAYS-NH2 | 9550,94 | ± | 3897,97 | 0,42 | ± | 0,31 |  | ND |  |
| start population | 20 | FYPGQWAYS-NH2 | 12904,34 | ± | 2320,55 | 0,64 | ± | 0,42 |  | ND |  |
| start population | 21 | AwPGQFAyS-NH2 | 16465,74 | ± | 12339,93 | 0,30 | ± | 0,34 |  | ND |  |
| start population | 22 | YWPGQWAfC-NH2 | 18646,47 | ± | 5762,07 | 0,44 | ± | 0,40 |  | ND |  |
| start population | 23 | WFPGQYAYS-NH2 | 20694,24 | ± | 7671,34 | 0,44 | ± | 0,37 |  | ND |  |
| start population | 24 | YfPGQfAfS-NH2 |  | NR |  |  |  |  |  | ND |  |
| start population | 25 | YFPGNFYFG-NH2 |  | NR |  |  |  |  |  | ND |  |
| start population | 26 | YFPGQfAFt-NH2 |  | NR |  |  |  |  |  | ND |  |
| start population | 27 | YFwGQFAFS-NH2 |  | NR |  |  |  |  |  | ND |  |
| 0 | 28 | YLPGQFfFS | 0,77 | ± | 0,37 | 0,97 | ± | 0,11 | 24 | ± | 3 |
| 0 | 29 | YFPGQFAFG | 0,79 | ± | 0,54 | 1,01 | ± | 0,22 | 13 | ± | 1 |
| 0 | 30 | YFPGQYAFF | 1,89 | ± | 0,9 | 0,98 | ± | 0,09 | 26 | ± | 8 |
| 0 | 31 | YFPGHFAFS | 6,63 | ± | 2,91 | 1,07 | ± | 0,12 | 17 | ± | 4 |
| 0 | 32 | YFPGNFAFS | 9,96 | ± | 4,42 | 0,89 | ± | 0,13 | 16 | ± | 2 |
| 1 | 33 | YLPGQFAFs | 1,72 | ± | 0,32 | 0,51 | ± | 0,28 | 31 | ± | 0 |
| 1 | 34 | FLPGQYAFS | 2,75 | ± | 0,14 | 0,68 | ± | 0,39 | 28 | ± | 1 |
| 1 | 35 | YVPGQFAFf | 4,17 | ± | 2,31 | 0,96 | ± | 0,16 | 42 | ± | 19 |
| 1 | 36 | YFpGQFAFS | 4,18 | ± | 3,53 | 1,01 | ± | 0,32 | 27 | ± | 1 |
| 1 | 37 | YFPGQFAFs | 4,21 | ± | 2,03 | 0,96 | ± | 0,01 | 23 | ± | 0 |
| 1 | 38 | YLPGQYAFL | 5,75 | ± | 2,56 | 1,05 | ± | 0,14 | 27 | ± | 4 |
| 1 | 39 | YLPGQFAFL | 5,85 | ± | 2,62 | 1,01 | ± | 0,13 | 24 |  |  |
| 1 | 40 | YMPGQFAFs | 6,26 | ± | 2,75 | 0,74 | ± | 0,36 | 23 |  |  |
| 1 | 41 | YLPGQFwFS | 7,23 | ± | 3,1 | 0,94 | ± | 0,16 | 45 |  |  |
| 1 | 42 | YLPGQFAfF | 8,93 | ± | 7,43 | 1,07 | ± | 0,08 | 30 |  |  |
| 1 | 43 | yLPGQYAFF | 9,23 | ± | 3,68 | 1,13 | ± | 0,04 | 135 | ± | 39 |
| 1 | 44 | YLPGQFQFS | 9,54 | ± | 4,19 | 0,76 | ± | 0,15 | 15 |  |  |
| 1 | 45 | YFPGqFAFS | 12,15 | ± | 4,86 | 0,88 | ± | 0,17 | 23 |  |  |
| 1 | 46 | FLPGQYAFL | 13,5 | ± | 3,13 | 1,06 | ± | 0,38 | 28 |  |  |
| 1 | 47 | yFPGQFAFS | 15,04 | ± | 8,54 | 0,95 | ± | 0,22 | 42 |  |  |
| 1 | 48 | YLPGQYAFY | 15,63 | ± | 7,44 | 0,92 | ± | 0,12 | 16 |  |  |
| 1 | 49 | YLPGQGAFP | 28,67 | ± | 12,17 | 0,97 | ± | 0,11 | 17 |  |  |
| 1 | 50 | YFPGQFAFQ | 52,64 | ± | 24,37 | 1,04 | ± | 0,07 | 17 |  |  |
| 1 | 51 | YFPGQFAfS | 64,23 | ± | 6,29 | 0,98 | ± | 0,08 | 16 |  |  |
| 1 | 52 | YFPGQFaFS | 75,93 | ± | 2,93 | 0,88 | ± | 0,25 | 17 |  |  |
| 1 | 53 | YFPGQFDFS | 84,81 | ± | 38,21 | 0,93 | ± | 0,25 | 16 |  |  |
| 1 | 54 | YfPGQFAFS | 88,29 | ± | 9,4 | 1,01 | ± | 0,27 | 52 |  |  |
| 1 | 55 | fLPGQYAFF | 92,87 | ± | 23,24 | 0,73 | ± | 0,24 | 230 |  |  |
| 1 | 56 | YLPGQYLFL | 113,51 | ± | 45,91 | 0,57 | ± | 0,34 | 19 |  |  |
| 1 | 57 | YLPGQFyFS | 118,61 | ± | 33,33 | 0,89 | ± | 0,16 | 26 |  |  |
| 1 | 58 | PLPGQfAFF | 285,51 | ± | 57,9 | 0,81 | ± | 0,17 | 33 |  |  |
| 1 | 59 | PGPGQFAFF | 335,48 | ± | 99,65 | 0,9 | ± | 0,07 | 80 |  |  |
| 1 | 60 | FLPGALGFF | 406,05 | ± | 114,95 | 0,69 | ± | 0,34 | 19 |  |  |
| 1 | 61 | FLPGQGfFS | 455,54 | ± | 165,46 | 0,73 | ± | 0,2 | 41 |  |  |
| 1 | 62 | YFPGQfAFS | 504,99 | ± | 299,41 | 0,9 | ± | 0,32 | 15 |  |  |
| 1 | 63 | FLPGQFLPG | 553,63 | ± | 298,99 | 0,81 | ± | 0,23 | 46 |  |  |
| 1 | 64 | FLPGQYAFK | 566,75 | ± | 177,48 | 0,93 | ± | 0,32 | 26 |  |  |
| 1 | 65 | FLPGQPYFL | 712,91 | ± | 86,03 | 0,91 | ± | 0,25 | 32 |  |  |
| 1 | 66 | FPPGQGAFK | 1181,3 | ± | 491 | 0,67 | ± | 0,48 |  | ND |  |
| 1 | 67 | FLPGQFAFK | 1244,05 | ± | 255,98 | 0,73 | ± | 0,23 | 16 |  |  |
| 1 | 68 | YPPGQYAFF | 1475,31 | ± | 420,95 | 0,94 | ± | 0,13 | 54 |  |  |
| 1 | 69 | YLPGQGAFK | 1868,93 | ± | 1038,05 | 0,99 | ± | 0,12 | 22 |  |  |
| 1 | 70 | PLPGQFQFK | 1916,29 | ± | 899,55 | 0,43 | ± | 0,16 | 27 |  |  |
| 1 | 71 | QLPGQFAfF | 2458,26 | ± | 801,63 | 0,66 | ± | 0,26 | 31 |  |  |
| 1 | 72 | FLPGFLALp | 2606,68 | ± | 986,2 | 0,64 | ± | 0,22 | 218 |  |  |
| 1 | 73 | FLPPQFAFF | 3001,19 | ± | 709,81 | 0,77 | ± | 0,36 | 20 |  |  |
| 1 | 74 | LLPGLFLKL | 5116,7 | ± | 884,76 | 0,85 | ± | 0,09 | 39 |  |  |
| 1 | 75 | YlpGQFLFS | 5581,32 | ± | 1267,28 | 0,99 | ± | 0,09 | 49 |  |  |
| 1 | 76 | YFLGQFAFK | 5833,57 | ± | 8110,34 | 0,65 | ± | 0,2 | 16 |  |  |
| 1 | 77 | YLPGYLPGH | 216845,21 | ± | 113287 | 1,04 | ± | 0,12 | 17 |  |  |
| 1 | 78 | YLPGQAfFK | 232621,85 | ± | 188912 | 1,15 | ± | 0,15 | 24 |  |  |
| 1 | 79 | PLPGQQAFF | 2033264,3 | ± | 1473699 | 0,6 | ± | 0,24 | 84 |  |  |
| 2 | 80 | YLPGQYAFf | 0,21 | ± | 0,22 | 1,77 | ± | 0,29 | 36 | ± | 4 |
| 2 | 81 | YLPGQYAFs | 0,4 | ± | 0,23 | 1,73 | ± | 0,39 | 22 | ± | 2 |
| 2 | 82 | yLPGQYAFS | 3,62 | ± | 2,91 | 1,61 | ± | 0,3 | 77 | ± | 5 |
| 2 | 83 | fLPGQYAFf | 8,2 | ± | 3,96 | 1,82 | ± | 0,48 | 1157 | ± | 41 |
| 2 | 84 | YRPGQYAFs | 8,62 | ± | 5,01 | 1,84 | ± | 0,44 | 21 | ± | 4 |
| 2 | 85 | yMPGQYAFS | 11,75 | ± | 6,05 | 1,78 | ± | 0,31 | 75 |  |  |
| 2 | 86 | YVPGQYAFs | 21,78 | ± | 12,32 | 1,57 | ± | 0,43 | 24 | ± | 8 |
| 2 | 87 | YLPGQYAYs | 27,46 | ± | 10,88 | 1,62 | ± | 0,35 | 49 |  |  |
| 2 | 88 | YyPGQYAFs | 35,12 | ± | 13,83 | 1,51 | ± | 0,26 | 891 |  |  |
| 2 | 89 | FLPGQFwFs | 49,25 | ± | 26,91 | 1,76 | ± | 0,31 | 73 |  |  |
| 2 | 90 | YVPGQFAFs | 49,97 | ± | 18,27 | 1,81 | ± | 0,47 | 23 | ± | 6 |
| 2 | 91 | yVPGQYAFS | 53,63 | ± | 27,81 | 1,58 | ± | 0,29 | 151 |  |  |
| 2 | 92 | YAFsQYAFs | 60,87 | ± | 29,1 | 1,54 | ± | 0,22 | 52 |  |  |
| 2 | 93 | YAFsPGQFs | 64,42 | ± | 30,71 | 1,67 | ± | 0,27 | 81 |  |  |
| 2 | 94 | YEPGQYAFs | 76,21 | ± | 39,54 | 1,56 | ± | 0,41 | 47 |  |  |
| 2 | 95 | fLPGQYAFs | 80,64 | ± | 40,7 | 1,75 | ± | 0,32 | 2437 |  |  |
| 2 | 96 | sHlGQYAdS | 90,52 | ± | 44,72 | 1,63 | ± | 0,4 | 3248 |  |  |
| 2 | 97 | PGpGQFAFs | 100,35 | ± | 28,51 | 1,72 | ± | 0,26 |  | ND |  |
| 2 | 98 | ylPGQFAFs | 106,82 | ± | 29,1 | 1,68 | ± | 0,32 | 1092 |  |  |
| 2 | 99 | VPGQYAFss | 128,72 | ± | 35,01 | 1,7 | ± | 0,32 | 43 |  |  |
| 2 | 100 | GAFQFsAFs | 129,47 | ± | 49,34 | 1,69 | ± | 0,25 | 71 | ± | 6 |
| 2 | 101 | yLPGQYAYS | 140,26 | ± | 22,37 | 1,56 | ± | 0,21 | 81 |  |  |
| 2 | 102 | QFsFAYAFA | 142,53 | ± | 34,35 | 1,78 | ± | 0,35 | 83 |  |  |
| 2 | 103 | FQFAFSAFs | 193,96 | ± | 83,79 | 1,76 | ± | 0,37 |  | ND |  |
| 2 | 104 | yLpGQYAFS | 245,89 | ± | 104,35 | 1,73 | ± | 0,4 | 80 |  |  |
| 2 | 105 | FPGQFAFss | 611 | ± | 316,63 | 1,81 | ± | 0,31 | 62 | ± | 16 |
| 2 | 106 | AFsQFsAFs | 696,63 | ± | 24,7 | 1,53 | ± | 0,22 | 110 |  |  |
| 2 | 107 | PSyVPGQYA | 1055,07 | ± | 344,61 | 1,97 | ± | 0,26 | 362 |  |  |
| 2 | 108 | fLYGLYAFp | 1214,5 | ± | 177,13 | 1,61 | ± | 0,17 |  | ND |  |
| 2 | 109 | FLFGFQFAY | 1654,81 | ± | 180,27 | 1,7 | ± | 0,27 | 16 |  |  |
| 2 | 110 | FLPGQFAFp | 1774,9 | ± | 183,56 | 1,59 | ± | 0,2 | 65 |  |  |
| 2 | 111 | PYVPLdYAF | 1939,83 | ± | 584,1 | 1,5 | ± | 0,31 | 92 | ± | 7 |
| 2 | 112 | yDPGQFAFs | 1985,89 | ± | 332,76 | 1,12 | ± | 0,08 | 4990 |  |  |
| 2 | 113 | cYFGQYAFs | 2191,48 | ± | 904,15 | 1,56 | ± | 0,14 | 29 |  |  |
| 2 | 114 | FAFGqFAFs | 2438,27 | ± | 407,8 | 1,24 | ± | 0,13 |  | ND |  |
| 2 | 115 | QFAFAFAFs | 2486,56 | ± | 985,68 | 1,2 | ± | 0,21 | 24 |  |  |
| 2 | 116 | FLFGpLALp | 4141,37 | ± | 1459,68 | 0,9 | ± | 0,17 |  | ND |  |
| 2 | 117 | FAFAFsFAY | 4446,44 | ± | 2336,96 | 0,87 | ± | 0,2 | 26 |  |  |
| 2 | 118 | fLpYALpfL | 21460,8 | ± | 12610,78 | 0,61 | ± | 0,05 |  | ND |  |
| 3 | 119 | YLPsQYAFf | 0,31 | ± | 0,37 | 1,62 | ± | 0,29 | 63 | ± | 13 |
| 3 | 120 | yLPsQYAFf | 0,49 | ± | 0,13 | 1,97 | ± | 0,13 | 1693 | ± | 291 |
| 3 | 121 | yLPsQYAFs | 2,9 | ± | 1,34 | 1,6 | ± | 0,39 | 1408 | ± | 85 |
| 3 | 122 | YLPGQYwFf | 3,24 | ± | 1,02 | 1,54 | ± | 0,3 | 159 | ± | 56 |
| 3 | 123 | YLPqQYAFf | 4,24 | ± | 1,87 | 1,74 | ± | 0,43 | 87 | ± | 19 |
| 3 | 124 | YLPaQYAFs | 4,42 | ± | 3,41 | 1,75 | ± | 0,37 | 110 | ± | 26 |
| 3 | 125 | YLPGQYApf | 14,32 | ± | 3,11 | 1,69 | ± | 0,29 | 42 |  |  |
| 3 | 126 | YLPGQYwPf | 20,99 | ± | 14,1 | 1,33 | ± | 0,48 | 47 |  |  |
| 3 | 127 | YLPwQYAFf | 35,63 | ± | 21,35 | 1,52 | ± | 0,18 | 124 |  |  |
| 3 | 128 | LwGGPYwYs | 38,69 | ± | 6,78 | 1,45 | ± | 0,24 | 480 |  |  |
| 3 | 129 | YRlGQYwdf | 41,71 | ± | 18,91 | 1,1 | ± | 0,31 | 32 |  |  |
| 3 | 130 | YLPpPYAFf | 43,54 | ± | 8,67 | 1,72 | ± | 0,52 | 215 |  |  |
| 3 | 131 | LwGGPYAdS | 53,18 | ± | 13,42 | 1,67 | ± | 0,44 | 551 |  |  |
| 3 | 132 | GLQqFYwGf | 55,74 | ± | 9,49 | 1,82 | ± | 0,39 | 117 |  |  |
| 3 | 133 | yFPGQYwFf | 67,4 | ± | 13,12 | 1,62 | ± | 0,28 | 20 |  |  |
| 3 | 134 | AFGfqSAYS | 69,36 | ± | 8,05 | 1,37 | ± | 0,11 | 10 | ± | 1 |
| 3 | 135 | YLPGqYALf | 72,17 | ± | 6,89 | 1,48 | ± | 0,4 | 40 |  |  |
| 3 | 136 | YLPGyYALf | 74,99 | ± | 9,59 | 1,4 | ± | 0,39 | 97 |  |  |
| 3 | 137 | fQYyPGQYY | 103,21 | ± | 10,08 | 1,42 | ± | 0,08 | 108 | ± | 22 |
| 3 | 138 | LfGpYGwsA | 103,78 | ± | 5,35 | 1,97 | ± | 0,33 | 637 | ± | 32 |
| 3 | 139 | SHlGQFwFs | 104,46 | ± | 24,49 | 1,55 | ± | 0,33 | 56 |  |  |
| 3 | 140 | sHlGQYAFf | 105,34 | ± | 20,02 | 1,49 | ± | 0,2 | 612 |  |  |
| 3 | 141 | YLPGQYAaf | 108,42 | ± | 12,78 | 1,73 | ± | 0,42 | 34 |  |  |
| 3 | 142 | yLPGwYAFf | 128,31 | ± | 11,22 | 1,55 | ± | 0,43 | 421 |  |  |
| 3 | 143 | yLPpPYFLf | 138,75 | ± | 47,6 | 1,62 | ± | 0,36 |  | ND |  |
| 3 | 144 | LLYwPYAKs | 144,92 | ± | 12,95 | 1,56 | ± | 0,11 | 81 |  |  |
| 3 | 145 | YLPfYLPGf | 149,9 | ± | 24,37 | 1,02 | ± | 0,3 | 40 |  |  |
| 3 | 146 | fQYaQYwFs | 170,88 | ± | 28,31 | 1,49 | ± | 0,19 | 2261 |  |  |
| 3 | 147 | YLPYPYpFf | 206,75 | ± | 28,27 | 1,36 | ± | 0,24 | 28 |  |  |
| 3 | 148 | YLPdfQYLP | 206,84 | ± | 28,51 | 1,74 | ± | 0,1 | 32 |  |  |
| 3 | 149 | PLqYPYcLf | 271,41 | ± | 15,34 | 1,51 | ± | 0,49 | 30 |  |  |
| 3 | 150 | YLPyqYAQf | 284,01 | ± | 48,49 | 1,35 | ± | 0,15 | 62 |  |  |
| 3 | 151 | YLPlGYApf | 297,73 | ± | 7,54 | 1,47 | ± | 0,59 | 91 |  |  |
| 3 | 152 | YLPpPfYLP | 302,06 | ± | 66,46 | 1,7 | ± | 0,73 |  | ND |  |
| 3 | 153 | PPLwQYAds | 312,69 | ± | 21,06 | 1,31 | ± | 0,11 | 86 |  |  |
| 3 | 154 | yyPsFFaFP |  | NR |  |  |  |  | 374 | ± | 37 |
